# Influence of Digitalis on Nutrition

**Published:** 1871-03

**Authors:** 


Influence of Digitalis on Nutrition.—M. M. A. Megevand has reported (*Gazette, Hebdom*, 12 Aug. 1870) a number of experiments instituted with a view of ascertaining the influence of digitalis on nutrition. He concludes from these that digitaline, and especially digitalis, notably diminishes the urea, and this diminution he considers to be closely connected with the slowing of the pulse of which it is the corollary, and, further, that it explains the antiphlogistic effects of the drug.—*Ibid.*

